# APLN/APLNR Signaling Controls Key Pathological Parameters of Glioblastoma

**DOI:** 10.3390/cancers13153899

**Published:** 2021-08-02

**Authors:** Roland E. Kälin, Rainer Glass

**Affiliations:** 1 Neurovascular Research, Department of Neurosurgery, University Hospital, LMU Munich, 81377 Munich, Germany; 2 Walter Brendel Center of Experimental Medicine, Faculty of Medicine, LMU Munich, 81377 Munich, Germany; rglass@med.lmu.de; 3 Neurosurgical Research, Department of Neurosurgery, University Hospital, LMU Munich, 81377 Munich, Germany; 4 German Cancer Research Center (DKFZ), German Cancer Consortium (DKTK), Partner Site Munich, 69120 Heidelberg, Germany

**Keywords:** Apelin (APLN), APLNR, Glioblastoma (GBM), tumor microenvironment (TME), tumor associated myeloid cells (TAM), pericytes, tumor parenchyma, vascular normalization, angiogenesis, invasion

## Abstract

**Simple Summary:**

The neurovascular peptide Apelin and its receptor APLNR are upregulated during glioblastoma pathology. Here we summarize their role in the brain tumor microenvironment composed of neurons, astrocytes, and the vascular and immune systems. Targeting APLN/APLNR signaling promises to unfold multimodal actions in future GBM therapy, acting as an anti-angiogenic and an anti-invasive treatment, and offering the possibility to reduce neurological symptoms and increase overall survival simultaneously.

**Abstract:**

Glioblastoma (GBM) is the most common and aggressive primary brain tumor in adults. GBM-expansion depends on a dense vascular network and, coherently, GBMs are highly angiogenic. However, new intratumoral blood vessels are often aberrant with consequences for blood-flow and vascular barrier function. Hence, the delivery of chemotherapeutics into GBM can be compromised. Furthermore, leaky vessels support edema-formation, which can result in severe neurological deficits. The secreted signaling peptide Apelin (APLN) plays an important role in the formation of GBM blood vessels. Both APLN and the Apelin receptor (APLNR) are upregulated in GBM cells and control tumor cell invasiveness. Here we summarize the current evidence on the role of APLN/APLNR signaling during brain tumor pathology. We show that targeting APLN/APLNR can induce anti-angiogenic effects in GBM and simultaneously blunt GBM cell infiltration. In addition, we discuss how manipulation of APLN/APLNR signaling in GBM leads to the normalization of tumor vessels and thereby supports chemotherapy, reduces edema, and improves anti-tumorigenic immune reactions. Hence, therapeutic targeting of APLN/APLNR signaling offers an interesting option to address different pathological hallmarks of GBM.

## 1. Introduction

Glioblastoma (GBM) is the most frequent and most aggressive primary brain tumor. GBM can originate from neoplastic neural precursor cells (NPCs) [1] after ablation or somatic mutation of the tumor suppressor p53 or loss of cdkn2a. These genetic aberrations can coincide with the loss of the phosphatase and tensin homolog deleted on chromosome-10 (PTEN). The vast majority of primary GBM is driven by a genetic mutation in key tumor suppressor genes concomitant with the accelerated activity of different proto-oncogenic signaling pathways (e.g., epidermal growth factor receptor, EGFR, or platelet- derived growth factor receptor-A, PDGFRA) or through a mutant (ligand-independent) form of EGFR (EGFR-variant-3, EGFRvIII) [1].

This genetic diversity drives different levels of tumor heterogeneity, which represent major caveats for successful GBM treatment [2,3]. Understanding the role of the tumor microenvironment (TME) can help to develop new therapies against GBM as it strongly supports neoplastic progression [4,5]. More than 30% of the cells in a GBM are brain parenchymal cells [6] and this GBM microenvironment consists of a complex mix of brain resident cells, such as microglia, astrocytes, and neurons as well as endothelial cells (ECs), pericytes of the neoplastic vasculature, and peripheral immune cells (monocytes, macrophages, and lymphocytes) [4,6,7,8]. The physiological role of these stromal components was intensively investigated and shown to support angiogenesis, GBM cell invasion, and proliferation as well as suppression of anti-tumor immunity [9,10]. The identification of signaling cues between tumor supporting and neoplastic cells can indicate novel therapeutic approaches against GBM.

In the last two decades, inhibition of vascular endothelial growth factor A (VEGFA) signaling to target tumor endothelia evolved as a new concept for tumor therapy [11]. However, VEGFA-inhibition failed to prolong overall survival in clinical trials for GBM therapy [12,13]. In the search for new pathways involved in GBM pathology, the mRNA of the angiogenic factor Apelin (APLN) and its G-protein coupled receptor, Apelin receptor (APLNR) were found to be upregulated in GBM-associated vascular proliferations as well as in hypoxic tumor regions [14], where co-expression with VEGFA was observed [14,15]. Today our knowledge on APLN/APLNR signaling in GBM cells and the brain TME has considerably advanced. We have gained important insight into APLN/APLNR-mediated effects in brain tumor associated neurons, astrocytes, the vasculature (consisting of ECs and pericytes), as well as the immune compartment (formed by tumor-associated myeloid cells and T-lymphocytes).

## 2. APLN and Its Receptor APLNR

The human APLNR gene (formerly known as APJ for putative receptor protein related to the angiotensin II receptor-like 1) was identified through its sequence homology to angiotensin II receptor type 1 (AT1R) [16,17]. The frog orthologue for APLNR (called Msr for a mesenchyme-associated serpentine receptor) was cloned thereafter and found to be expressed in blood vessels of venous as well as arterial origin [18]. Based on these findings, APLNR (Msr/APJ) was established as a reliable endothelial marker in embryonic cardiovascular development of Xenopus tadpoles [14,19,20] and subsequently of rodents [21,22,23,24].

In a ligand screen for orphan G-protein coupled receptors (GPCR), Tatemoto and colleagues [25] identified a small bioactive peptide ligand for APJ (APLNR) that was termed Apelin (for APJ endogenous ligand). From the peptide sequence, they cloned the human and bovine Apelin (APLN) cDNA that encoded for a secreted preprotein of 77 amino acids (Apelin-77) [25,26]. Upon proteolytic maturation, Apelin is further processed into its bioactive isoforms including Apelin-36, Apelin-17, Apelin-13, and the pyroglutamylated (Pyr1) Apelin-13 [25]. Interestingly, the most C-terminal 14 amino acids containing the Apelin-13 peptide remained 100% identical from amphibians to humans throughout evolution [14].

## 3. APLN/APLNR in Vascular Development

During embryogenesis, APLNR was found to be expressed throughout the developing vasculature while its ligand APLN was localized to the leading edge of APLNR-positive vessels (e.g., in the retina) [18,21]. The close structural relationship of APLNR to CXC chemokine receptors suggested Apelin as a chemotactic signal for ECs [27]. Subsequently, this was experimentally confirmed for APLNR-expressing ECs in vitro [14,28,29]. In a series of functional experiments in vivo, we and others could demonstrate that APLN signaling is necessary and sufficient to promote angiogenic sprouting during embryonic development [14,29]. APLN expression was found to be induced by VEGF signaling [14] and hypoxia due to a hypoxia-responsive element in the APLN gene promoter [29,30]. On initial inspection, APLN knockout (KO) mice were reported to exhibit mild vascular effects such as reduced vessel diameter, compared to wild-type (WT) controls, while APLN overexpressing mice developed enlarged but stable vessels with reduced vascular permeability [31,32]. Later, reassessment of APLN-KO mice demonstrated retardation of retinal vascular development [30]. Thus, Apelin appears to act in a paracrine fashion first to stimulate angiogenic sprouting and second in an autocrine manner to sustain endothelial motility via the angiogenic tip cells, where endothelial APLN is most strongly expressed [14,31,33,34,35]. Consequently, APLN-creER reporter mice receiving adequate tamoxifen stimulation allow to readily differentiate sprouting endothelium from stabilized vasculature during development and in pathology [36].

## 4. APLN in the Formation of the Glioblastoma Vasculature

**Figure 1:**
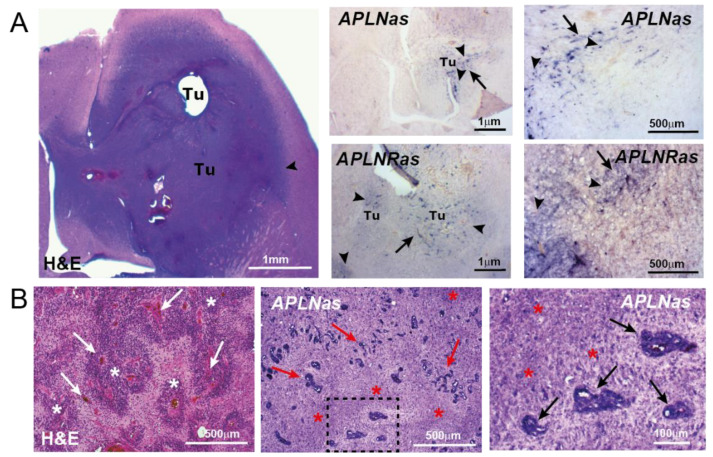
APLN/APLNR upregulation in primary GBM. In situ hybridization (ISH) against APLN or APLNR mRNA was performed on horizontal sections in a GBM implantation model (**A**) using murine proneural-like p53^KO^PDGFB GBM cells [42] or in patient primary GBM samples (**B**). (**A**) The picture on the left shows the aggressively invasive tumor (Tu) as stained by H&E, the arrowhead points to the invasive tumor border. The pictures on the right indicate APLN or APLNR mRNA (in pink) in tumor vessels (arrows) and tumor cells (arrowheads) using APLN/APLNR antisense ISH probes. (**B**) Patient biopsies were stained by H&E and APLN ISH was performed on consecutive sections. Note that APLN expression is high in vascular proliferates (arrows) and in the neighboring hypoxic areas of pseudopalisading necroses (asterisks). Micrographs of Figure 1B are adapted from Mastrella et al., 2019 [42]. Scale size is indicated in individual micrographs.

An extensive, aberrant vascularization is one of the hallmarks of GBM [37]. We detected a dramatic upregulation of APLN and APLNR mRNA expression in the GBM-associated microvascular proliferations [14]. Together with a comparative study of gene expression profiles in tumor versus normal endothelium [38], this was the first indication for an angiogenic role of APLN in human tumors. APLNR was later identified as part of an angiogenic gene signature in more than 1000 different well-vascularized primary human cancer biopsies [39]. Another indication for a central role of APLN/APLNR signaling in neoplastic vascularization came from a serial xenograft model recapitulating the angiogenic switch in GBM [40,41]. Here, vascular expression levels of APLN and APLNR mRNA increased concomitant with a switch from an invasive to an angiogenic histopathological GBM phenotype [42]. Using adult APLN-creER [36] and APLNR-creER [43] transgenic mouse models, it was confirmed that APLN/APLNR expression is low in adult physiology but it is upregulated in ECs during tumor angiogenesis [36]. To test the impact of APLN derived from the TME, APLN-KO mice were used in various orthotopic GBM models (using syngeneic tumor cells or xenografts) [42,44]; consistently, APLN was found to be upregulated in the tumor neovasculature as compared to tumor-free control areas (Figure 1A). In contrast, orthotopic implantation of GBM cells into APLN-KO mice [45] resulted in significantly reduced GBM angiogenesis as compared to WT controls [42,44]. These experiments highlighted intratumoral ECs as one major source for APLN in neoplasia and suggested that autocrine APLN/APLNR signaling in ECs co-controls tumor angiogenesis.

In addition to the strong expression in tumor vessels, APLN was also detected in GBM pseudopalisades (Figure 1B), which represent another hallmark of this entity and are formed by radially oriented neoplastic cells surrounding band like necroses [14,42]. The role of tumor-cell derived APLN was investigated by shRNA-mediated APLN knock-down (APLN-KD), which demonstrated that depletion of APLN expression from tumor cells also decreased tumor vessel density (independently from host-derived APLN). Importantly, combining APLN-KO and APLN-KD had synergistic anti-angiogenic effects and reduced pathological vascularization in a GBM model to a level comparable to (or even lower than in) healthy brain regions; infusion of the Apelin-13 peptide was able to rescue the APLN loss-of-function phenotype [44]. Experiments performed in mouse models of lung and mammary tumors confirmed the contribution of paracrine and autocrine APLN to tumor angiogenesis [46]. In these tumor models, APLN expression levels directly correlated with the rate of angiogenesis and survival [44]. This is in line with experiments where ectopic overexpression of APLN in subcutaneous tumor implants led to increased tumor vessel formation and enhanced tumor growth [47].

## 5. The Role of APLN and APLNR in Glioblastoma Cell Invasion

Another therapeutically challenging feature of GBM is the highly infiltrative growth pattern of this tumor [37]. Invading GBM cells can express high levels of APLNR, and this elevated expression correlates with increased expression of genes involved in tumor cell invasion like MMP2 or BAI1/3 [42]. In contrast, high APLN expression levels were restricted to tumor cell-dense and highly vascularized areas of the main tumor mass. Experimentally reducing APLN expression in orthotopic (syngeneic or xenograft) models for GBM led to increased tumor invasiveness [42].

Stimulation of APLNR was additionally reported to support GBM stem cell maintenance [48]. Recently, a second ligand for APLNR, named APELA (for Apelin receptor early endogenous ligand), was identified [49,50] and found to be present in GBM samples [51]. Specifically, APELA expression was localized to the brain stem cell niches in nestin-positive cells. Thus, APLEA could be involved in gliomagenesis, acting as a mitotic factor for neoplastic tumor initiating NPCs [1].

APLN and APLNR are known to be expressed in the developing mesoderm [14,18,21,29] and there is increasing literature on APLNR expression in pluripotent stem cells, such as hematopoietic stem cells (HSC), playing a role in HSC maturation and maintenance [52,53]. As GBM stem cells were previously shown to possess the capacity to trans-differentiate into ECs [54,55], the question arises if the angiogenic APLN peptides may be involved in this process. Although APLNR is expressed in GBM stem cells at variable levels [42,48], such a connection still needs to be shown.

In embryonic stem cells (ESC), a specific mechanism for APELA function in stem cell maintenance was proposed. Interestingly, APELA mRNA was shown to modulate Tp53 induced apoptosis [56]. In ESCs, p53 activity needs to be kept in check because unwanted activation of p53 will cause the differentiation and/or apoptosis of ESCs. In their study, the authors found that APELA RNA levels were significantly higher in murine p53-KO than in p53-WT ESCs and that p53 actively repressed APELA expression by identified p53-binding sites on its enhancer region. Hence, they tested if APELA might be involved in p53-mediated stress response. Interestingly, they found that APELA RNA binds to heterogeneous nuclear ribonucleaoprotein L (hnRNPL), an inhibitory regulator of p53. They found that APELA promoted damage-induced p53-dependent apoptosis by this tri-element negative feedback loop. This indicates that APLNR ligand expression can have unexpected roles and may also contribute to genetic integrity.

Both, APLN and APELA peptides are known to signal through APLNR by inhibitory Gi proteins [25,49,57,58], leading to adenylyl cyclase inhibition [58,59] and an increase of intracellular calcium concentrations [60]. Eventually, this can lead to the activation of extracellular-regulated kinases (ERKs) [57], which are critically involved in cell division and often deregulated during oncogenesis [61]. In several different cell types, APLN and APELA were also described to signal through the PI3K/akt pathway [62,63,64], another important driver for gliomagenesis [3].

## 6. Apelin-F13A Blocks Glioblastoma Invasion and Simultaneously Attenuates Tumor Angiogenesis

Our studies demonstrated that VEGFA and APLN are co-expressed in GBM hypoxic areas [14,15], that blockade of VEGFA/VEGFR2 signaling (using bevacizumab or ramucirumab) downregulates APLN [65], and that blockade of VEGFA/VEGFR2 and attenuation of APLN-levels synergistically reduce tumor angiogenesis but also increased tumor cell invasion [42,66]. Strikingly, co-application of the partial APLNR antagonist Apelin-F13A [67] together with VEGF/VEGFR blocking antibodies reduced both angiogenesis and GBM-invasion [42]. Others confirmed the anti-angiogenic effect of the Apelin-F13A peptide in peripheral tumors after systemic application, displaying no obvious side effects [43]. Moreover, a novel bi-cyclic peptide MM54 [68] with increased in vivo stability was applied systemically and resulted in reduced vascularization and increased survival in a GBM mouse model [48]. A study on mammary and lung cancer mouse models targeting APLNR with the antagonist MM54 was also successful in reducing blood vessel density, and significantly attenuated the treatment side effects of the receptor tyrosine kinase inhibitor Sunitinib (blocking VEGFR2 kinase) by reducing the metastatic spread of tumor cells to the lungs [46]. Together, these studies indicate that APLNR blockade acts synergistically with VEGFA-blocking anti-angiogenesis by attenuating infiltrative GBM growth and decreasing resistance to current anti-angiogenic therapies.

## 7. APLN and APLNR in Neurons and Astrocytes

In addition to the high expression levels found in tumor cells during pathology [42,44], low but specific APLNR immunoreactivity can also be detected in certain discrete brain regions under physiological conditions, which include pyramidal neurons in the striatum and the cortex, the hypothalamic paraventricular and supraoptic nuclei, the pituitary, the pineal gland, and Purkinje cells in the cerebellum [22,24]. Based on this expression pattern, an involvement of APLNR signaling in a range of neurophysiological processes such as in the regulation of hormone release, circadian rhythm, and water and food intake was suggested [22,24]. In addition, a neuroprotective role of APLN/APLNR signaling was reported for hippocampal and cortical neurons [69].

**Figure 2:**
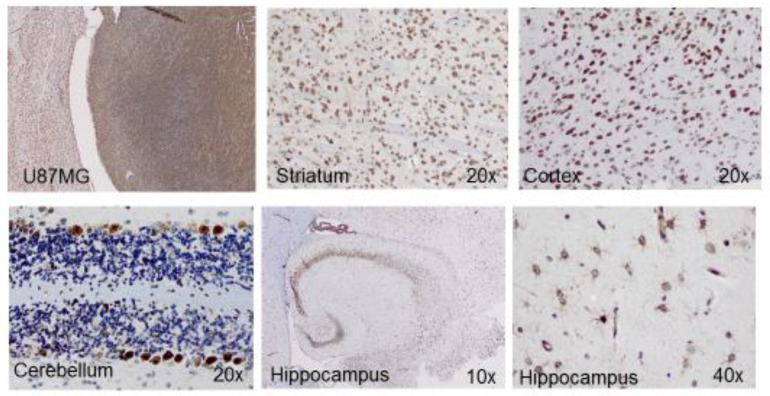
APLNR expression in neurons and astrocytes of the GBM-bearing mouse. Expression levels of APLNR in a healthy brain are compared to the immunoreactivity found in U87MG tumor cells. Magnifications and the region of the healthy brain structures are indicated in the individual panels.

By immunohistological analysis of APLNR in tumor-bearing mice, we could confirm such a neuronal expression pattern (Figure 2). In addition, we found APLNR immunoreactivity in astrocytes of the hippocampus (Figure 2) and the reactive gliosis surrounding tumors in mouse GBM models [70]. Previously, APLNR was detected in cultured astrocytes [60] and a functional role of APLN/APLNR signaling in astrocyte maturation during retinal angiogenesis was described [71]. Here, the authors demonstrated that APLNR- and APLN-deficient mice have delayed retinal angiogenesis but contain aberrant endothelial networks with immature astrocytes. In neuropathology, reactive astrocytes form a protective barrier that limits the extent of tissue damage, contributing to the process of wound healing in the brain (e.g., by repairing the blood-brain-barrier (BBB)) [72,73]. If APLN signaling contributes to this reparative process remains undetermined.

## 8. APLN and APLNR in Pericytes

Pericytes are vascular cells positioned in the basement membrane of blood vessels, closely attached to brain capillary ECs [74]. By integrating signals from neighboring cells in the so-called neurovascular unit (ECs, astrocytes, and neurons), they ensure proper CNS function by maintaining the integrity of the BBB and stabilization of the vessel architecture [75]. Furthermore, pericytes regulate capillary tone and diameter [76,77].

APLN/APLNR signaling contributes to the regulation of vascular tone, as shown after intravenous injection of APLN peptide [63,78]. Support for APLN as an active player in blood pressure regulation comes from patients with essential hypertension in which circulating levels of Apelin-12 were significantly lower [79]. That APLN is a potent endothelium-dependent vasodilator was previously shown [80]. Double mutant mice, lacking both APLNR and angiotensin-1 receptor (AT1R), had a higher baseline blood pressure than mice lacking AT1R only, suggesting a counter-regulatory role of APLN to that of AT1 in blood pressure regulation [81]. Interestingly, central administration of (Pyr1)Apelin-13 in rats caused an increase in arterial blood pressure [82], indicating that APLN is likely more important in central than in peripheral regulation of the cardiovascular system.

Reassessment of the vascular phenotype in APLN knockout mice indicated a change in vessel diameter to more narrow blood vessels, while APLN-overexpressing mice showed enlarged but stable vessels with reduced vascular permeability [31,32]. In line with that, inhibition of APLN in a mouse model for retinopathy showed a change in pericyte coverage [83]. Qin et al. (2013) found that ectopic expression of APLN led to increased pericyte to EC ratios, as shown in a tube formation assay in vitro and a murine hindlimb ischemia model in vivo [84]. In a subcutaneous tumor model, APLN overexpression reduced the leakiness of the tumor vasculature [85]. Expression of APLNR was detected in human cardiomyocytes and vascular smooth muscle cells by immunocytochemistry [86]. Immunohistological proof for APLN/APLNR expression in pericytes was also described in vitro after hypoxia in patients with diabetic retinopathy [87]. Thus, it is conceivable that APLN/APLNR signaling in pericytes may effect vascular maturation and control vessel tightness. It remains to be shown if Apelin and its receptor also control tightness of the blood tumor barrier in GBM.

## 9. APLN/APLNR Signaling in Microglia, Macrophages, and T-Cells

Microglia are sessile macrophages of the brain, which control innate immunity and contribute to an adaptive immune response in the CNS [88]. Microglia, together with bone-marrow-derived macrophages, are commonly termed tumor-associated myeloid cells (TAM) [4,6]. TAM density in glioma directly correlates with malignancy, invasiveness, and grading of the tumor [89,90,91]. It was shown that TAM could promote tumor expansion and accelerate GBM cell invasion [7,8,92]. They can secrete pro-tumorigenic factors like TGFβ, IL6, and EGF [8], driving immunosuppression, but can also act pro-angiogenic by supporting, for example, vascular anastomosis in the tumor [93,94,95]. Transgenic, bone marrow reconstituted GBM mouse models assigned such a direct proangiogenic effect specifically to microglia rather than to monocyte-derived macrophages [96]. Recently, we demonstrated that tumor-associated cells with a myeloid-like expression profile (TAMEP), which express a range of myeloid markers but do not derive from microglia or the bone marrow, largely control tumor angiogenesis and GBM-progression [97]. The high cell density of TAM in GBM is caused by a variety of factors secreted by the tumor cells that can attract TAM, namely CCL2, CX3CL1, CXCL12, CSF1, or VEGFA [98]. Kerber et al. (2008) [99] demonstrated that VEGFA overexpression in glioma xenografts led to a massive infiltration of monocytes/macrophages. Moreover, the loss of FLT1 function in monocytes in vitro abrogated VEGFA-induced chemotaxis. However, in vivo FLT1 loss-of-function did produce the opposite effect. Thus, it seems that additional factors regulate the inflammatory infiltration of the xenografts, with monocytes/macrophages shaping the heterogeneity of the TME. In this context, it is important to note that, in recurrent GBM, the TAM composition shifts towards a higher ratio of monocyte-derived macrophages over microglia, in correlation with an increase in aggressive invasiveness [100,101].

Interestingly, our gene ontology analysis of APLN co-regulated genes in GBM genetic subtypes revealed that high APLN expression was associated with vascular morphogenesis in the proneural and classical subtypes, but not in the mesenchymal subtype [42]. This finding corresponds with the observation that the proneural and classical GBM subtypes respond better to anti-angiogenic therapy than mesenchymal GBM [102,103]. Comparing expression data of GBM patients from the TCGA database, we found that genes co-regulated with APLNR fell into Gene-Clusters primarily correlating with an anti-tumor immune response (Table 1). Such a direct immunomodulatory effect was previously described for APLN/APLNR signaling by Leeper and colleagues (2009) [104] in a mouse model for vascular disease. Here, infusion of Apelin peptide into mice prevented aortic aneurysm formation by inhibiting macrophage recruitment. At the site of inflammation, macrophage burden was lowered and pro-inflammatory cytokine production for TNFA, IL-6, CCL2, and CSF1 was attenuated. In a different context, ectopic Apelin expression in the skin led to a reduction of UVB-induced edema and a decrease in the number of CD11b-positive macrophages [105].

Contradicting results exist concerning APLNR expression in microglia and macrophages. While two studies reported no expression of APLNR RNA and protein in cultured human primary microglia and blood monocyte-derived macrophages [60,106], a recent study demonstrated that murine monocytes and several cell lines of mouse macrophages not only express APLNR mRNA but also react to APLN. A possible explanation for these discrepancies could be an upregulation of APLN/APLNR upon macrophage activation. Following the findings of Leeper et al. (2009) [104], we also found APLNR to be expressed in CD11b-MACS sorted microglia from the brains of mice (unpublished observation).

In a mouse implantation model for mammary carcinoma, the complete loss of APLN expression from tumor cells (by APLN-KD) and the TME (using APLN-KO) led to a decrease in tumor vessel density and tumor volumes [46]. Assessment of the immune cell compartment showed that numbers of CD11b-positive inflammatory monocytes were not changed in these models. Treating experimental GBM models with Apelin-F13A had anti-angiogenic and anti-invasive effects and reduced tumor volume but did not lead to a decrease in TAM [42]. This may indicate that APLNR blockade is not immune suppressive in GBM, but this remains to be investigated in more detail. In their study of peripheral tumors, Uribesalgo et al. (2019) [46] found myeloid-derived suppressor cells to be decreased and NK-cells to be increased upon APLN depletion. In our gene cluster analysis for APLNR coregulated genes in GBM, the top annotation clusters also indicated a role for APLN/APLNR signaling in lymphocyte-mediated and NK-mediated immunity (Table 1). Altogether, these data suggest a role for APLN as an immune-regulatory factor in GBM and other tumors.

That the APLN/APLNR signaling system is present in T-lymphocytes is known, since 1998, when APLNR (APJ) was identified as a co-receptor for HIV entry into T-cells [107,108]. Further functional evidence for its involvement in the regulation of the adaptive immune system was found when Apelin suppressed cytokine production from mouse spleen cells in response to T cell receptor/CD3 cross-linking [58]. Finally, evidence for the role of APLN/APLNR signaling in adaptive immunity came from a recent study that identified APLNR as one of the genes essential for immunotherapy in cancer [109]. The authors describe that APLNR expression in the tumor augments T^eff^ cell function by increasing interferon γ (INF γ) signaling and CTLA-4 blockade efficiency, supporting the effectiveness of T-cell based therapy. APLN/APLNR signaling seems thus to play a role in the modulation of the immune system in general, but the impact of the described effects in GBM must be further elucidated.

## 10. Perspectives for APLN-Mediated Multimodal Glioblastoma Therapy

A common feature of primary CNS tumors is the formation of tumor-associated brain edema that will eventually lead to neurological symptoms [110]. Corticosteroids are the most commonly used agents in the management of tumor-associated brain edema [111,112]. Despite the application of the corticosteroid dexamethasone (DEX) for brain edema resolution, clinical attention is now raised on potential side effects including abnormal glucose metabolism, leukopenia, and pneumonia infections [113]. DEX is also applied upon tumor recurrence. Regardless of its obvious effects to alleviate the symptoms from cerebral vasogenic edema, the overall corticosteroid exposure appears to be an independent risk factor for lymphopenia-associated reduction in overall survival [114]. Furthermore, there is evidence that DEX can have cytoprotective effects on glioma cells in vitro [115] and compromise survival in glioblastoma models in vivo [116]. In other tumor entities, the promotion of cancer metastasis by glucocorticoids was also observed [117]. Corticosteroids induce apoptotic cell death in lymphocytes and may have unwanted effects on tumor-associated macrophages or myeloid-derived suppressor cells in GBM [115,118,119]. Edema management with DEX can thus counteract immunotherapies, including chimeric antigen receptor (CAR) T cells, vaccines, and immune checkpoint blockers [120,121,122,123,124]. Out of these reasons, there is a growing need to find alternatives for DEX therapy for edema management in GBM patients [120].

**Figure 3:**
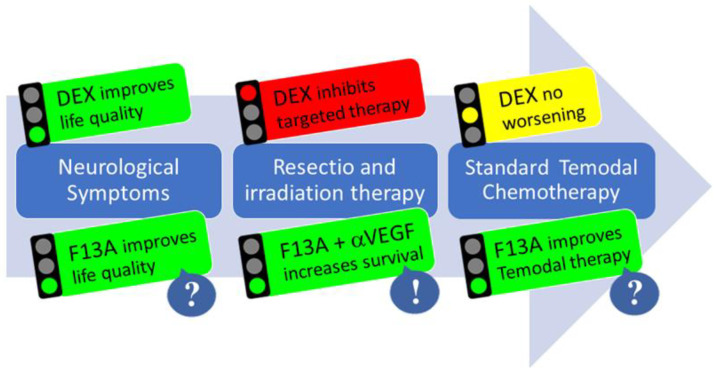
The multimodal action of APLN/APLNR targeting therapy in GBM. In phase 1 of the disease, DEX treatment improves neurological symptoms; after resection, DEX may reduce options for targeted therapy (e.g., immunotherapy) in phase 2. In phase 3, during chemotherapy and tumor recurrence, DEX shows no change. For Apelin-F13A (F13A) therapy, there is evidence that it could improve life quality by reducing edema, that anti-angiogenic combination treatment moderately increases survival, and that vascular normalization could improve temozolomide chemotherapy.

Here, anti-VEGF therapy has gained some value in clinical practice to (transiently) control edema and as a substitute for corticosteroids [125]. However, the potentially adverse effects of bevacizumab-treatment (e.g., accelerated invasion [126]) remain. Modulation of APLN/APLNR signaling by Apelin-F13A represents a potentially more favorable option for edema management [85], as this pathway co-controls vascular integrity and may attenuate vasogenic edema in GBM (without promoting invasion). Finally, co-treatment with bevacizumab and Apelin-F13A may augment vascular normalization and improve intratumoral delivery of chemotherapeutics [109] (Figure 3).

## 11. Conclusions

In summary, recent research on APLN/APLNR signaling in tumor pathology indicates that pharmacological modulation of the APLN/APLNR pathway can act as anti-angiogenic and anti-invasive treatment. Moreover, APLN inhibition seems to further reduce the proliferation of glioma stem cells and confer neuroprotection in the brain. In addition, targeting APLN/APLNR signaling offers the unique possibility to reduce neurological symptoms and to increase overall survival. Hence, targeting APLN/APLNR signaling with a BBB-permeable small chemical compound holds the promise that this single drug could unfold multimodal actions in future GBM therapy.

## Figures and Tables

**Table 1:** APLNR expression in GBM correlates with gene ontology clusters involved in inflammatory immune response (marked in green). Functional DAVID annotation clustering using co-regulated genes (919 with a Pearson coefficient of >0.25).

Annotation Cluster	Representative Annotation Term	Enrichment Score
1	immune (acute inflammatory) response (lymphocyte-mediated immunity)	5.31
2	keratinization/epidermal cell differentiation	2.94
3	Natural killer cell-mediated cytotoxicity (autophagy, Toll-like receptor/JakSTAT/interferon signaling)	2.63
4	lysosome/lytic vacuole	2.23
5	ATP binding	2.19
6	Helicase and RNase D C-terminal, HRDC	2.19
7	G1/S transition of mitotic cell cycle/interphase	2.07
8	BRCT	2.06
9	nuclear division/cell division/M phase of mitotic cell cycle	1.93
10	SH3	1.91
11	ATP-dependent helicase activity	1.88
12	MHC class II protein complex/antigen processing and presentation	1.78
13	integral to plasma membrane	1.73
14	T cell selection and differentiation/leukocyte activation	1.70
15	endocytosis/membrane invagination	1.67

## Abbreviations

APJ	putative receptor protein related to the angiotensin II receptor-like 1 (Angtrl1)
APLN	Apelin
APLN-KD	APLN knockdown
APLN-KO	APLN knockout mouse
APLNR	Apelin receptor
BBB	blood-brain barrier
creER	cre recombinase estrogen receptor fusion gene
DEX	Dexamethasone
ECs	endothelial cells
ESCs	embryonic stem cells
GBM	glioblastoma
GPCR	G-protein-coupled receptor
HE	Hematoxylin/Eosin
HSC	hematopoietic stem cells
KD	knockdown
TAM	tumor associated cells
TME	tumor microenvironment
Msr	mesenchyme-associated serpentine receptor
NPCs	neural precursor cells
VEGFA	Vascular endothelial growth factor A
VEGFR	VEGF receptor
WT	wildtype mouse
